# Specialized structural and functional roles of residues selectively conserved in subfamilies of the pleckstrin homology domain family

**DOI:** 10.1002/2211-5463.12725

**Published:** 2019-09-30

**Authors:** Nagarajan Naveenkumar, Ramanathan Sowdhamini, Narayanaswamy Srinivasan

**Affiliations:** ^1^ National Centre for Biological Sciences TIFR Bangalore India; ^2^ Bharathidasan University Tiruchirappalli India; ^3^ Molecular Biophysics Unit Indian Institute of Science Bangalore India

**Keywords:** Arf6, ARNO, GRK2, multidomain proteins, PH domain, phosphoinositide binding

## Abstract

Homologous domains embedded in multidomain proteins of different domain architectures (DA) may exhibit subtle, but important, differences in their structure and function. Here, we consider two multidomain proteins, Arf nucleotide binding site opener (ARNO) and G protein‐coupled receptor kinase 2 (GRK2), which have very different DAs, but both contain pleckstrin homology (PH) domains. We analyzed the roles of residues selectively conserved in these subfamilies of PH domains from ARNO and GRK2 proteins. DA‐specific residues in PH domain are found to contribute to structural and functional specialization of ARNO and GRK2 in terms of (a) specific intra‐ and interprotein interactions; (b) specificity for phospholipids; and (c) participation in conformational excursions, leading to various functional forms. Our approach can also be applied to subfamilies of other protein families to identify subfamily‐specific residues and their specialized roles.

**Abbreviations**
Arf6: ADP‐ribosylation factor 6
ARNO: Arf nucleotide binding site opener
DA: domain architecture
GRK2: G protein‐coupled receptor kinase 2
PH: pleckstrin homology domain

Homologous proteins are known to perform same or similar functions, but their functional levels, specificity to ligands, and regulatory mechanisms may vary among homologues 1. These differences between homologues are known to be achieved by their sequence differences and the kinds of domains to which the domain of interest is tethered in multidomain proteins 2. Domain tethering has been shown to influence the functional levels of the constituent domains 3. In this paper, we have investigated the roles of residues with certain conservation features in homologous domains from two multidomain proteins. The multidomain proteins with homologous domains differ in their sequential order of domain families [domain architectures (DA)]. We have classified the sequences of homologous protein domains into two subfamilies based on their DA and identified the residues that are selectively conserved in a subfamily corresponding to a DA. This work has been pursued for two different subfamilies of pleckstrin homology (PH) domains that are ADP‐ribosylation factor (Arf) nucleotide binding site opener (ARNO) and G protein‐coupled receptor kinase 2 (GRK2). We find that many of the subfamily‐specific residues of PH domain are interacting with adjacent domains in the multidomain protein, thus providing an explanation for their subfamily‐specific nature and functional specialization at the level of subfamilies.

Pleckstrin homology domain is named after the protein pleckstrin, which is found in platelets 4. PH domains are known to bind to lipids and also known for localizing proteins to the membrane 5. This domain has also been implicated in various protein–protein interactions 6. A typical PH domain averages 120 amino acids in length, and its structure consists of two antiparallel beta sheets packed against each other with a C‐terminal helix. Members of PH domain family are known for different ligand binding sites and for specificity for different ligands 6.

Pleckstrin homology domain is very versatile in nature and is known to be tethered with many protein domains 6. It is also noted that the PH domains themselves retain large sequence variation. Proteins sharing PH domains, but with varying DAs, were chosen and analyzed further. The proteins analyzed in this work are ARNO and GRK2. The DAs of these proteins are shown in Fig. 1. The roles of the residues in PH domains in these proteins that are selectively conserved within a PH domain subfamily corresponding to a specific DA are explored using the available 3D structures of these proteins in the Protein Data Bank 7 and known information on functional residues.

**Figure 1:**
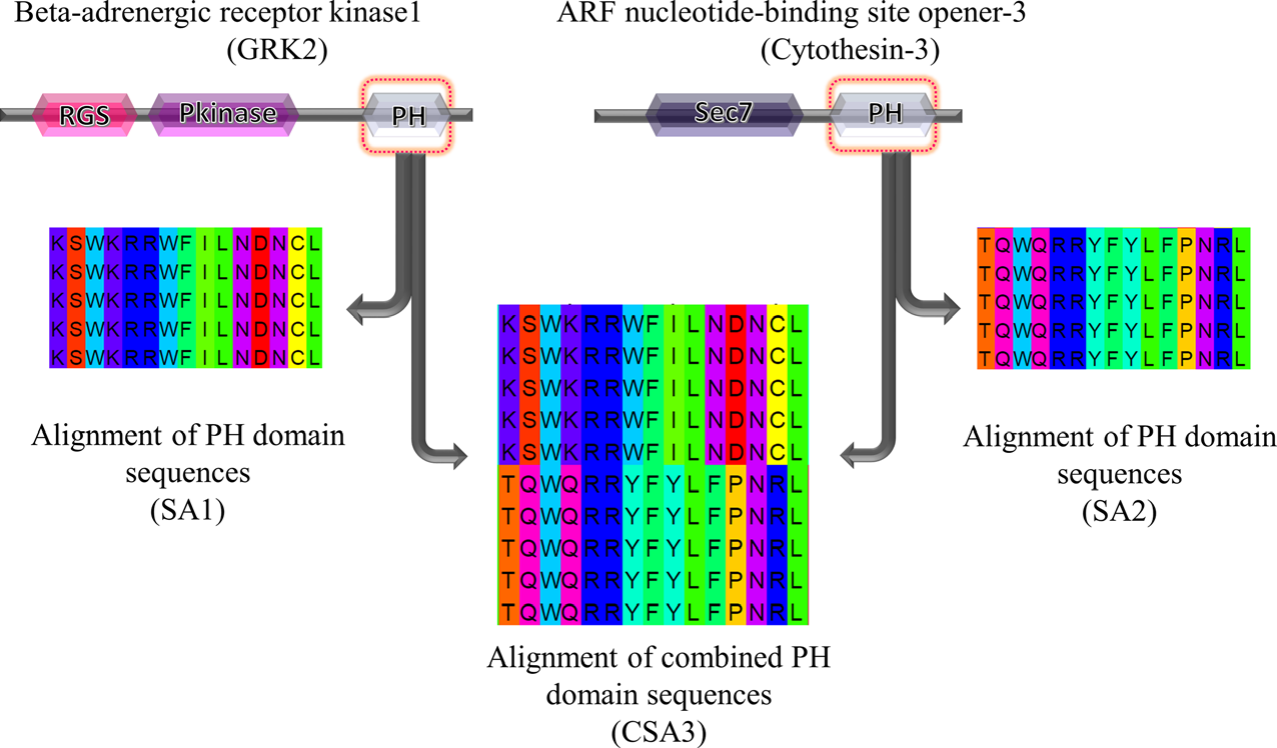
Recognition of DA‐specific residues in PH domains occurring in ARNO and GRK2. SA1 is a multiple SA of PH domain sequences of GRK2 homologs. SA2 is a multiple SA of PH domain sequences of ARNO homologs. CSA3 multiple SA of PH domain sequences of ARNO and GRK2.

Arf nucleotide binding site opener protein consists of two structural domains, a Sec7 domain followed by a PH domain as shown in Fig. 1. The ARNO is also called as guanine nucleotide exchange factor since it activates Arf GTPase from its inactive GDP‐bound form to its active GTP‐bound form 8, 9, 10.

ARNO protein exists in three structural forms 11, 12, and these are referred to as inactive, partially active, and active forms in this article. These forms are characterized by closed and open nature of catalytic and activator sites. One of the major differences between the three conformers is the conformation and orientation of the C‐terminal helix.

Auto‐inhibited state is maintained by a self‐regulatory mechanism, wherein the major differences between them are the conformations and hinge dynamics 11, 12. In contrast, the active state is maintained by GTP‐bound Arf 6‐dependent allosteric mechanism. Arf6 protein interacts at the activator site comprising of the PH domain and proximal autoinhibitory elements (Sec7‐PH linker and C‐terminal helix). Upon allosteric activation, catalytic site of ARNO is released from its auto‐inhibited state.

There are two isoforms of ARNO that have been crystallized and structure determined in full‐length form 13, 14. These two isoforms are differentiated by the presence or absence of a single glycine residue in the β1/β2 loop of the PH domain 13, 14 and play a critical role toward phosphoinositide specificity. The isoforms are called as diglycine variant and triglycine variant will henceforth be referred as ARNO‐2G and ARNO‐3G, respectively. ARNO‐2G is known to bind to PtdIns (3,4,5) P3, and ARNO‐3G is known to bind to both PtdIns (3,4,5) P3 and a diphosphoinositide PtdIns (4, 5) P2. However, the binding affinity of ARNO‐3G with PtdIns (3,4,5) P3 is lower compared to ARNO‐2G 15, 16, 17.

GRK2 protein has three structural domains, namely RGS, protein kinase, and PH domain as shown in Fig. 1. GRK2 is involved in agonist‐induced desensitization of the beta‐adrenergic receptors 18, 19. It is involved in interaction with Gβγ subunits of heterotrimeric G protein via its PH domain 20, 21, 22. GRK2 protein is known to interact with multiple phosphoinositides like PtdIns (3, 4) P2 and PtdIns (4, 5) P2 23, 24, 25, 26, 27, 28, but its 3D structure in phosphoinositide bound form is not yet available. Both GRK2 and ARNO proteins are known to bind to the phosphate head group of phospholipid bilayer via its PH domains to perform their respective functions.

In this work, we have classified the PH domain sequences into two subfamilies based on their occurrence in ARNO and GRK2 proteins, which are characterized by different DAs. We recognize residues in PH domain that are selectively conserved in one subfamily compared to the other and explore their structural and functional roles.

## Materials and methods

The two datasets used in the analysis are sequences of homologous ARNO proteins and sequences of homologous GRK2 proteins with their respective DAs conserved within ARNO and within GRK2 homologues.

### Identification of homologues with a given domain architecture

Pfam database 29 has been used to identify homologous ARNO and GRK2 proteins. A Pfam database file contains the alignment of Pfam domains of all UniProt sequences (Pfam‐A.full.uniprot.gz updated 2018), and it is downloaded from Pfam FTP site. The full‐length sequences of homologues were obtained from UniProt 30.

A script was needed that uses the DAs of ARNO and GRK2 proteins as a query and searches in the Pfam database to identify homologous proteins with same DA. This script should be such that it seeks to avoid entries with incomplete/partial domains and long insertions between domains that may correspond to a domain that is yet to be recognized and documented in the domain databases. Therefore, we developed our own script to recognize PH domain containing sequences corresponding to two different DAs with the other desired features mentioned above. The Perl scripts developed are provided in Appendix S1. The Pfam domain boundary information was mapped on the hits to form the datasets of sequences of homologous proteins with DAs corresponding to ARNO and GRK2. A relaxation of 20 amino acid residues was allowed for the N, C terminus extensions and linker regions to account for insertions and deletions among homologues. The boundary relaxation for the domains was given based on the shortest and the longest domains observed in Pfam seed alignment. Protein fragments and obsolete entries were excluded. Hits with more than 90% sequence identity were identified using CD‐HIT 31 ( http://weizhongli-lab.org/cdhit_suite/cgi-bin/index.cgi?cmd=cd-hit) and are removed from the list of sequences for analysis to result in nonredundant datasets corresponding to two different DAs. We safely used a 90% sequence identity cutoff as some of the identical proteins show < 100% sequence identity due to additional few residues in one compared to the other either in N‐terminal end or C‐terminal end or both.

The dataset has been further refined by removing proteins with overlapping domains, incomplete domains, and domains with long insertions.

### Identification of domain architecture‐specific residues from the sequence alignment

The PH domain region of homologous sequences of ARNO and GRK2 was multiply aligned as depicted in Fig. 1. Another combined multiple sequence alignment (SA) of the PH domain alone (including PH domain sequences from both ARNO and GRK2 homologues) has been made. The alignments were performed using MAFFT algorithm 32 (version V7.394). The combined alignment is referred here as combined SA‐3 (CSA3), and the other two separate alignments of homologous PH sequences of GRK2 and ARNO were referred as SA1 and SA2, respectively, as explained in Fig. 1. Alignment was visualized using jalview 33 (version 2.10.3b1). The master alignment is presented in Fig. S1.

The conserved residue columns or conservatively substituted residue columns between the ARNO homologs and GRK2 homologs in the master alignment (CSA3) were identified, and its corresponding residue columns in SA1 and SA2 with more than or equal to 85% conservation have been considered as DA‐specific residue columns. A conserved residue column in SA1, but with a semi‐conservative or % residue conservation > 50 in its corresponding residue column with same residue or residue type in SA2 and vice versa was not considered to be DA‐specific residues. Intraprotein and protein–protein interactions were identified, from the Protein Data Bank entries 2r09, 4kax of ARNO and 5ukl of GRK2 proteins and using Protein Interactions Calculator 34. A criterion of solvent accessibility of ≤ 7% was used to identify buried residues, and naccess 35 (version 2.1.1) was used to calculate solvent accessibility. Polar contacts and water bridges between PH domain and phosphoinositide were identified from PDB entries 1u29 and 1u27 and visualized using pymol 36 (version 1.8.4.0). The residue numbering used in the figures and tables corresponds to their sequence extracted from the PDB entry. All the figures showing 3D structures in the article are made using Pymol 36.

## Results and Discussion

### Domain architecture‐specific residues from the sequence alignment

The final dataset consists of 88 homologous sequences of ARNO and 21 homologous sequences of GRK2.

The conserved residue columns or conservatively substituted residue columns in the PH domain region of ARNO homologs and GRK2 homologs with the cutoffs described in the Methods have been considered as DA‐ or subfamily‐specific (Fig. 1). Henceforth, such residues will be referred as DA‐specific residues.

Further, solvent‐buried residues of PH domain of ARNO and GRK2 were identified as described in Methods and the corresponding aligned residue columns were not considered for further analysis, as buried residues are generally known to be situated in the core maintaining the tertiary structural integrity of the domain.

There are 16 of the alignment positions in the PH domain that are conserved or conservatively substituted across the members of ARNO and GRK2. As these are conserved across all the PH domain sequences from ARNO and GRK2 subfamilies, these are not DA‐specific residues. Out of these, 10 of the residues are also buried and hence could be contributing to the structural integrity of the domain fold. Two other residues, Trp‐281 and Trp‐285, maintain the conformation of the inositol binding pocket. Similarly, residues Lys‐273 and Gly‐275 interact with phosphoinositide and its equivalents are also part of the binding site region for phosphoinositide 21.

### Selectively conserved or conservatively substituted residues within ARNO members and within GRK2 members

If a residue in an alignment position is conserved only within ARNO members or only within GRK2 members in the datasets, then such residues are considered to be DA‐specific.

Using this criterion, 40 DA‐specific residues were identified for ARNO PH domains and 21 DA‐specific residues for GRK2 PH domains. If in an alignment position different residue types are conserved within ARNO (say Ser) and within GRK2 (say Val), these residues also qualify as DA‐specific residues for ARNO and GRK2, respectively.

The structural and functional roles of DA‐specific residues for both ARNO and GRK2 are provided in Tables 1 and 2, respectively.

**Table 1:** Roles of DA‐specific residues of PH domain in ARNO

Residue and number	Functional role (Interdomain interactions)	Functional role (Interaction with Arf‐6)	Functional role (phosphoinositide interactions)
Asp‐266	Interacts with N‐terminal helix and Sec7‐PH linker to maintain inactive form of ARNO. Similarly interacts with Sec7 ‐PH linker to maintain partially active form of ARNO		
Arg‐267	Interacts with Sec7‐PH linker to maintain inactive form of ARNO		
Glu‐268	Aids in maintaining the partially active form of ARNO		
Gly‐276			Interacts with phosphoinositides, 4IP and I3P
Arg‐277			Interacts with phosphoinositides, 4IP and I3P
Lys‐279			Maintains PH domain and phosphoinositide interaction of both 4IP and I3P
Thr‐280			Interacts with phosphoinositides, 4IP and I3P
Lys‐282			Interacts with phosphoinositides, 4IP and I3P
Ile‐287		Maintains interaction with Arf‐6 protein	
Thr‐289	Interacts with Sec7‐PH linker to maintain inactive form of ARNO	Maintains interaction with Arf‐6 protein	
Asp‐290	Interacts with Sec7‐PH linker to maintain inactive form of ARNO	Interacts with Arf‐6 protein	
Cys‐292	Maintains the inactive form of ARNO	Interacts with Arf‐6 protein	
Tyr‐294		Interacts with Arf‐6 protein	Maintains PH domain and phosphoinositide I3P interactions
Tyr‐295			Interacts with phosphoinositides, 4IP and I3P
Phe‐296		Maintains interaction with Arf‐6 protein	Maintains PH domain and phosphoinositide I3P interactions
Glu‐297			Maintains PH domain interactions with both 4IP and I3P phosphoinositides
Asp‐301			Maintains the conformation of loop3, where loop3 is known to act as phosphoinositide specificity determinant
Lys‐302			Maintains the conformation of loop3, where loop3 is known to act as phosphoinositide specificity determinant
Glu‐303			Maintains PH domain and phosphoinositide I3P interaction
Pro‐304		Interacts with Arf‐6 protein	Maintains PH domain and phosphoinositide I3P interaction
Pro‐309		Interacts with Arf‐6 protein	
Ile‐315	Maintains both inactive and partially active forms of ARNO		
Arg‐316	Role unknown		
Val‐318	Role unknown		
Lys‐323	Role unknown		
Tyr‐330	Role unknown		
Lys‐340		Interacts with Arf‐6 protein	
Ala‐341		Maintains interaction with Arf‐6 protein	Maintains PH domain and phosphoinositide I3P interaction
Cys‐342		Interacts with Arf‐6 protein	Interacts with phosphoinositide I3P
Lys‐343			Interacts with phosphoinositides 4IP and I3P
Thr‐344			Interacts with phosphoinositide 4IP and maintains PH domain and phosphoinositide I3P interaction
Glu‐345			Interacts with phosphoinositide 4IP and maintains PH domain and phosphoinositide I3P interaction
Asp‐347			Maintains PH domain and phosphoinositide 4IP interaction
Gly‐348			Maintains PH domain and phosphoinositide 4IP interaction
Glu‐352			Interacts with phosphoinositides 4IP and I3P
His‐355			Interacts with phosphoinositides 4IP and I3P
Arg‐359			Interacts with phosphoinositides 4IP and I3P
Glu‐366	Role unknown		
Met‐372	Role unknown		
Ile‐379	Interacts with Sec7‐PH linker to maintain both inactive and partially active forms of ARNO		

**Table 2:** Roles of DA‐specific residues in PH domain of GRK2

Residue and number	Functional role (Interdomain interactions)	Functional role (Protein–protein interaction)	Functional role (phosphoinositide interactions)	Functional role (Other functions)
Met‐561	Interacts with N‐terminal helix			
His‐562	Interacts with N‐terminal helix and maintains kinase‐PH linker and PH interactions			
Pro‐571			Maintains the conformation of loop1 for phosphoinositide binding	
Phe‐572			Maintains the conformation of loop1 for phosphoinositide binding	
Thr‐574			Maintains the conformation of loop1 for phosphoinositide binding	
Gln‐577			Maintains the phosphoinositide interaction	
Phe‐584	Interacts with Kinase‐PH linker			
Pro‐585	Maintains kinase‐PH linker and PH interactions	Interacts with Gβγ protein		
Arg‐587	Interacts with kinase and PH linker	Interacts with Gβγ protein		
Glu‐589	Interacts with kinase and PH linker	Interacts with Gβγ protein		
Gly‐592			Present in loop3 region, which is known to be involved in specificity determination	
Glu‐593			Present in loop3 region which is known to be involved in specificity determination	
Ile‐614			Interacts with loop1 region and maintains PIP2 binding	
Lys‐615			Interacts with loop1 region and maintains PIP2 binding	
Leu‐621	Role unknown			
Lys‐623	Role unknown			
Arg‐625	Interacts with C‐terminal extension	Maintains C‐terminal extension and Gβγ protein interaction		
Asp‐637	Interacts with RGS‐kinase linker		Critical for maintaining PIP2 binding pocket structure, because mutation affects PIP2 binding	
Leu‐640				Part of hydrophobic core and maintains the structure
Ala‐654		Interacts with Gβγ protein		
Leu‐657	Interacts with C‐terminal extension	Maintains C‐terminal extension and Gβγ protein interaction		
Val‐658	Interacts with C‐terminal extension	Maintains C‐terminal extension and Gβγ protein interaction		

### Roles of some of the DA‐specific residues in ARNO PH domains in inactive and partially active conformations

As mentioned before, the ARNO protein exists as three different conformers and Arf6 protein can interact with ARNO protein only to its partially active form, whereas it is inhibited in inactive form. This switch between inactive and partially active forms is maintained mainly by PH domain and its proximal regions in the protein 11, 12. Here, we have explained the roles of DA‐specific residues that are involved in specific interactions of different structural forms of ARNO; therefore, we believe these residues contribute to the stability of different conformational states of ARNO. Eight DA‐specific residues from PH domain of ARNO are involved in such interactions (Table 1 and Fig. S2). Although the residues Glu‐268 and Ile‐315 are not part of PH‐Sec7 interface in ARNO, they are considered to be important, since they are involved in interaction with domain–domain interface residues.

Six DA‐specific residues (Asp‐266, Arg‐267, Thr‐289, Arg‐290, Cys‐292, and Ile‐379) are involved in domain–domain interactions in the inactive form of ARNO, whereas two DA‐specific residues (Asp‐266 and Ile‐379) are involved in partially active form of ARNO (Table 1; Fig. S2). Residue Asp‐266 is a common interface residue in both the forms of ARNO, but confers differences in the mode of interaction. In the inactive form, this residue interacts with two residues (Tyr‐385 and Asn‐264 via hydrogen bond), but it interacts with only Asn‐264 (by hydrogen bond) in the partially active form of ARNO. In contrast to Asp‐266, residue Ile‐379 retains common interaction in both forms of ARNO. Thus, it is evident that a number of domain–domain interface residues in the two forms of protein play roles in the stability of domain–domain association.

### C‐terminal helix orientation: critical to maintain inactive and partially active forms of ARNO

**Figure 2:**
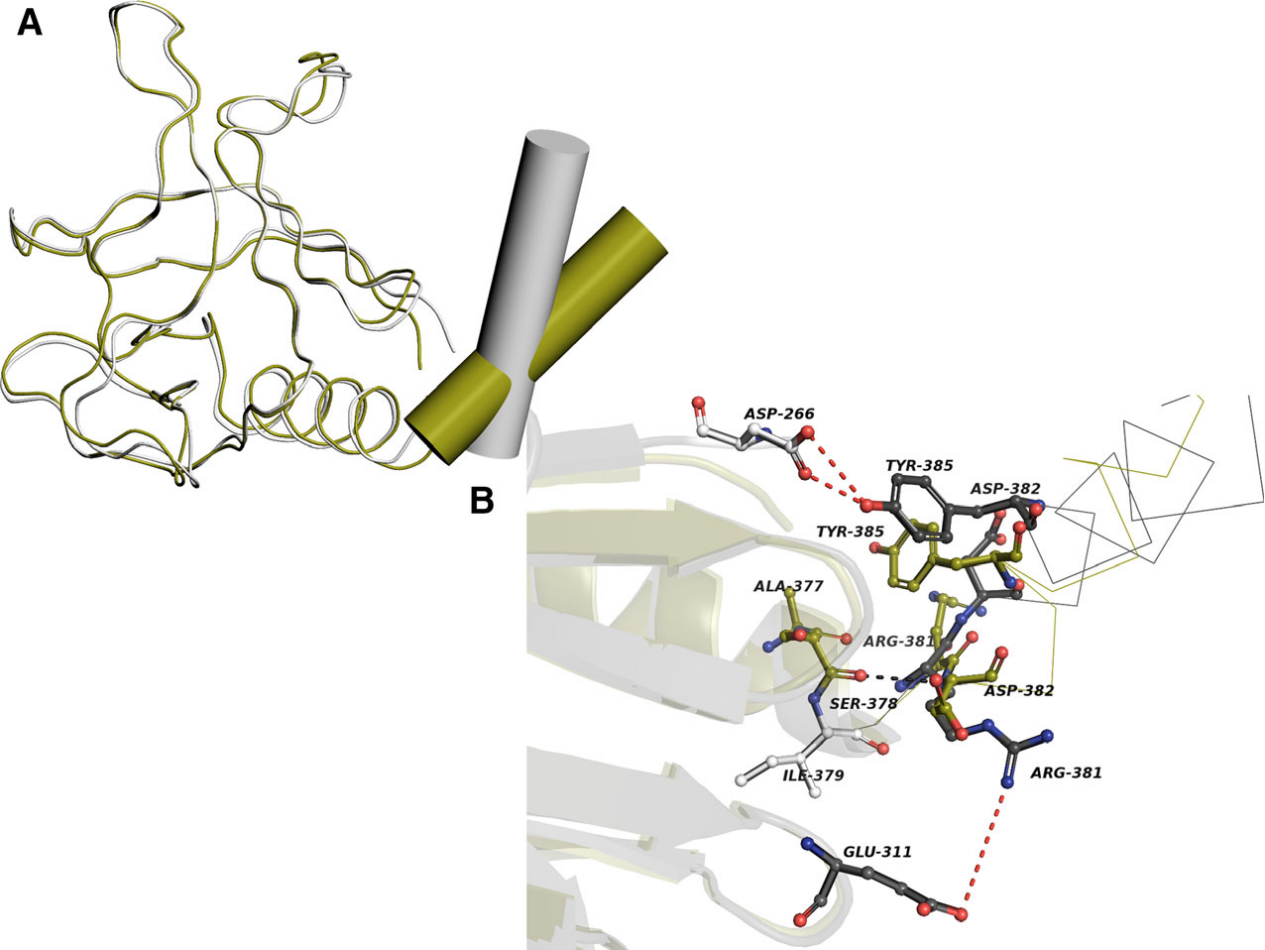
C‐terminal helix orientation in inactive and partially active forms of ARNO. (A, B) show the superposition of PH domains and C‐terminal helix in inactive and partially active forms of ARNO, and (B) is also the zoomed in view of PH domain and C‐terminal helix interface. The partially active form of ARNO is colored in green, and inactive form of ARNO is colored in gray. The residues playing roles in helix re‐orientation are shown in sticks. DA‐specific residues are colored in white. The interactions are shown in dotted lines, and the interactions maintained by DA‐specific residues are highlighted in red color.

One of the major features in determining the ARNO functional forms is the C‐terminal helix. The C‐terminal helix undergoes change in its orientation between the functional forms of ARNO. In inactive form, this helix interacts closely with PH domain and maintains a steric hindrance for Arf6 interaction for its interaction with PH domain. In the partially active form of ARNO, this C‐terminal helix undergoes a change in its orientation, thereby permitting Arf6 to interact with PH domain 12. Details of the DA‐specific residues involved in the helix re‐orientation are shown in Fig. 2.

The conformation and interactions of some of the residues play a role in the helix orientation as shown in Fig. 2B. Main chain–main chain hydrogen bond interaction between Ser‐378 and Asp‐382 seems related to helix orientation. This interaction is maintained in the partially active form and lost in inactive form of ARNO. The DA‐specific residue Ile‐379 is adjacent to Ser‐378 and plays an important role in maintaining this interaction. Other residues, involved in the re‐orientation of helix, are Arg‐381 and Tyr‐385. The conformation of Arg‐381 in the inactive form is characterized by a salt bridge between Arg‐381 and Asp‐311. Similarly, the conformation of Tyr‐385 (another DA‐specific residue) in its inactive form is characterized by hydrogen bond interactions with Asp‐266.

### DA‐specific residues involved in phosphoinositide binding in ARNO

The PH domain of ARNO binds to the cell membrane by mediating interaction with phospholipid head and helps to recruit other proteins to the membrane. The splice variant 2G of ARNO protein is known to interact with Ins (1,3,4,5) P4 13, but the other splice variant 3G with additional glycine insertion in the β1/β2 loop region of PH domain, and becomes dual‐specific 14. In addition to Ins (1,3,4,5) P4, it can also bind to another phosphoinositide Ins (1,4,5) P3. The terminologies used in PDB for Ins (1,3,4,5) P4 and Ins (1,4,5) P3 are 4IP and I3P, respectively.

There are nine DA‐specific residues involved in interaction with Ins (1,3,4,5) P4 in 2G variant and seven DA‐specific residues from 3G variant to interact with Ins (1,4,5) P3. Although both phosphoinositides bind in the same binding pocket, with six DA‐specific residues common between them, it does not imply that the DA‐specific residues are playing same roles. The two phosphoinositides bind in slightly different orientations, resulting in differences in interaction pattern. Despite being the part of same binding pocket, the DA‐specific residues interact with the phosphoinositides in different ways, explaining their vital role in providing specificity toward phosphoinositides.

Figure S3A,B show 2G variant of ARNO PH domain interaction with 4IP(or) Ins (1,3,4,5) P4 via hydrogen bonds and water bridges. The interactions are shown in separate figures for the sake of clarity. Figure S3C shows the 3G variant PH domain interaction with I3P by hydrogen bonds and water bridges.

### Roles of DA‐specific residues in Arf6 interaction: relieving auto‐inhibited state of ARNO

The partially active form of ARNO in its auto‐inhibited state allows Arf6 to interact with activator site. Upon accessing the activator site in PH domain and its proximal elements, it relieves the auto‐inhibited state of ARNO through allosteric activation. ARNO, upon interaction with Arf6, gets activated and turns from auto‐inhibited state to fully active state 12. Here, we explored the role of DA‐specific residues in Arf6 and PH domain complexation (Fig. S4). Apart from involving in direct protein–protein interaction, there are certain residue determinants that maintain the conformation of interface residues by intraprotein interaction. In ARNO, many DA‐specific residues are involved in multiple functions (see Table 1 for details).

**Figure 3:**
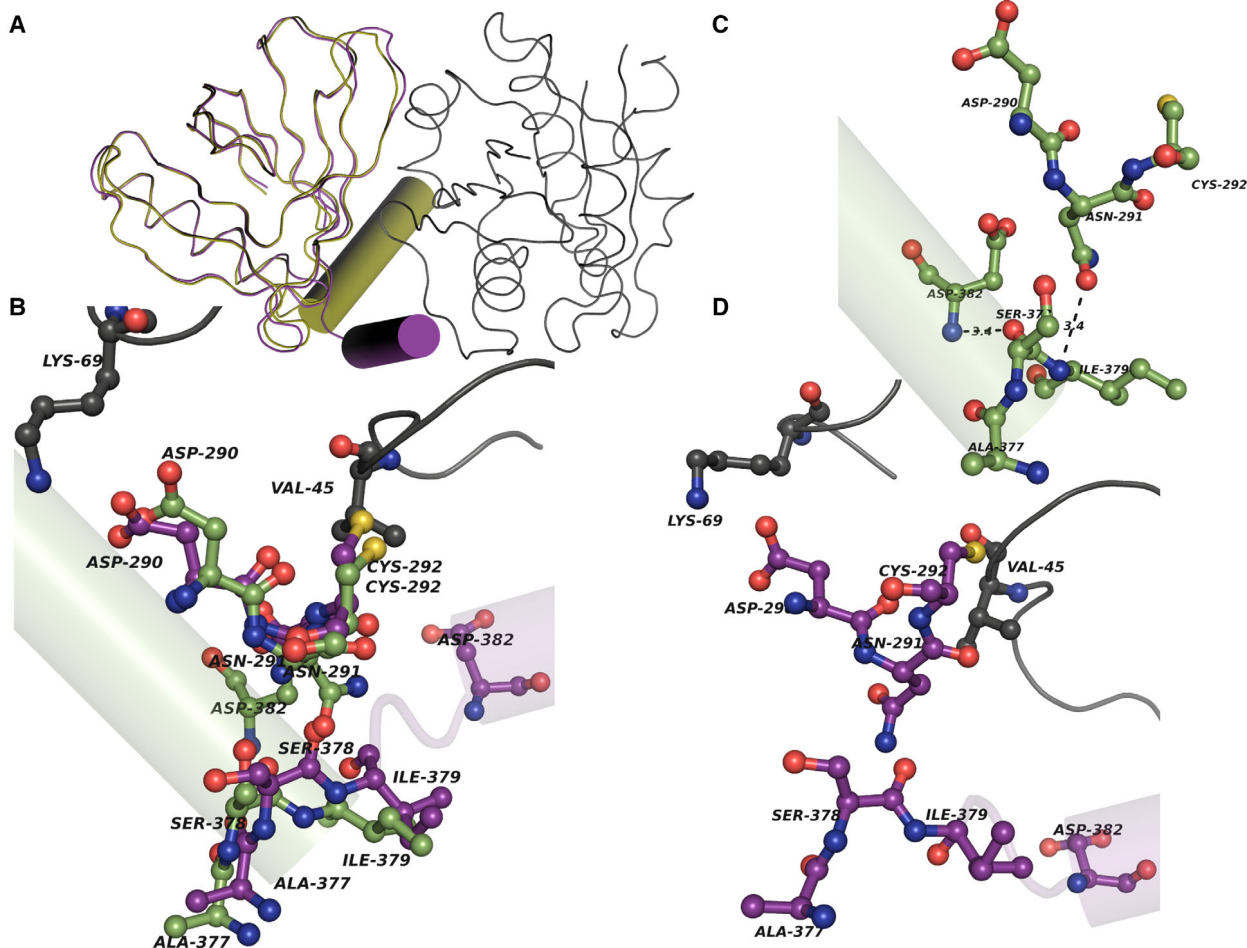
Superposition of the partially active and active conformers of ARNO. (A, B) show the superposition of PH domains and C‐terminal helix in partially active and active forms of ARNO (Arf6 bound form), and (B) also shows the conformation change in a series of residues between partially active and active forms of ARNO contributing to re‐orientation of C‐terminal helix. Active form is shown in complex with Arf6 highlighted in dark gray. The partially active form of ARNO is colored in green, and active form of ARNO is colored in purple. The residues are shown in ball and sticks, and the interactions are shown in dotted lines. (C) shows the interaction in connection with helix conformation in partially active form. (D) Conformational change in hinge residues, allowing re‐orientation of helix in active form of ARNO upon Arf6 interaction.

Arf6 interaction with PH domain seems to bring allostery‐mediated conformational change in ARNO. As mentioned already, re‐orientation of C‐terminal helix is noticed in the transition between inactive and partially active forms of ARNO. C‐terminal helix undergoes further change in orientation between partially active form (auto‐inhibited state) and active form of ARNO as shown by superposition of the two conformers of ARNO (Fig. 3A). Along with C terminus, few other conformational change in PH domain leads to release of catalytic site (active form) for substrate protein to interact (GDP‐bound Arf1). Helix re‐orientation occurs by a structural change in series of residues as shown by superimposed partially active conformer and active conformer in Fig. 3B. It is evident that Lys‐69 and Val‐45 of Arf6 play a critical role for C‐terminal helix re‐orientation in ARNO by direct interaction with ARNO residues as shown in Fig. 3B. We explored the roles of DA‐specific residues in such allosteric mechanism.

Loss of main chain–main chain hydrogen bond between Asp‐382 and Ser‐378 and main chain–side chain hydrogen bond between Asn‐291 and Ile‐379 seem to be related to re‐orientation of the helix (Fig. 3C,D). Overall, 8 interactions between unique residue pairs are lost between partially active form and active form that are involved in re‐orientation of C‐terminal helix.

As mentioned earlier, conformation of Asn‐291 and Ser‐378 seems to play roles in C‐terminal helix re‐orientation and is regulated by their adjacent and interacting DA‐specific residues Asp‐290, Cys‐292 and Ile‐379. Similarly, other DA‐specific residues such as Arg‐267 and Asp‐266 are also directly involved.

### Roles of DA‐specific residues in GRK2 PH domain in interdomain and interprotein interactions

**Figure 4:**
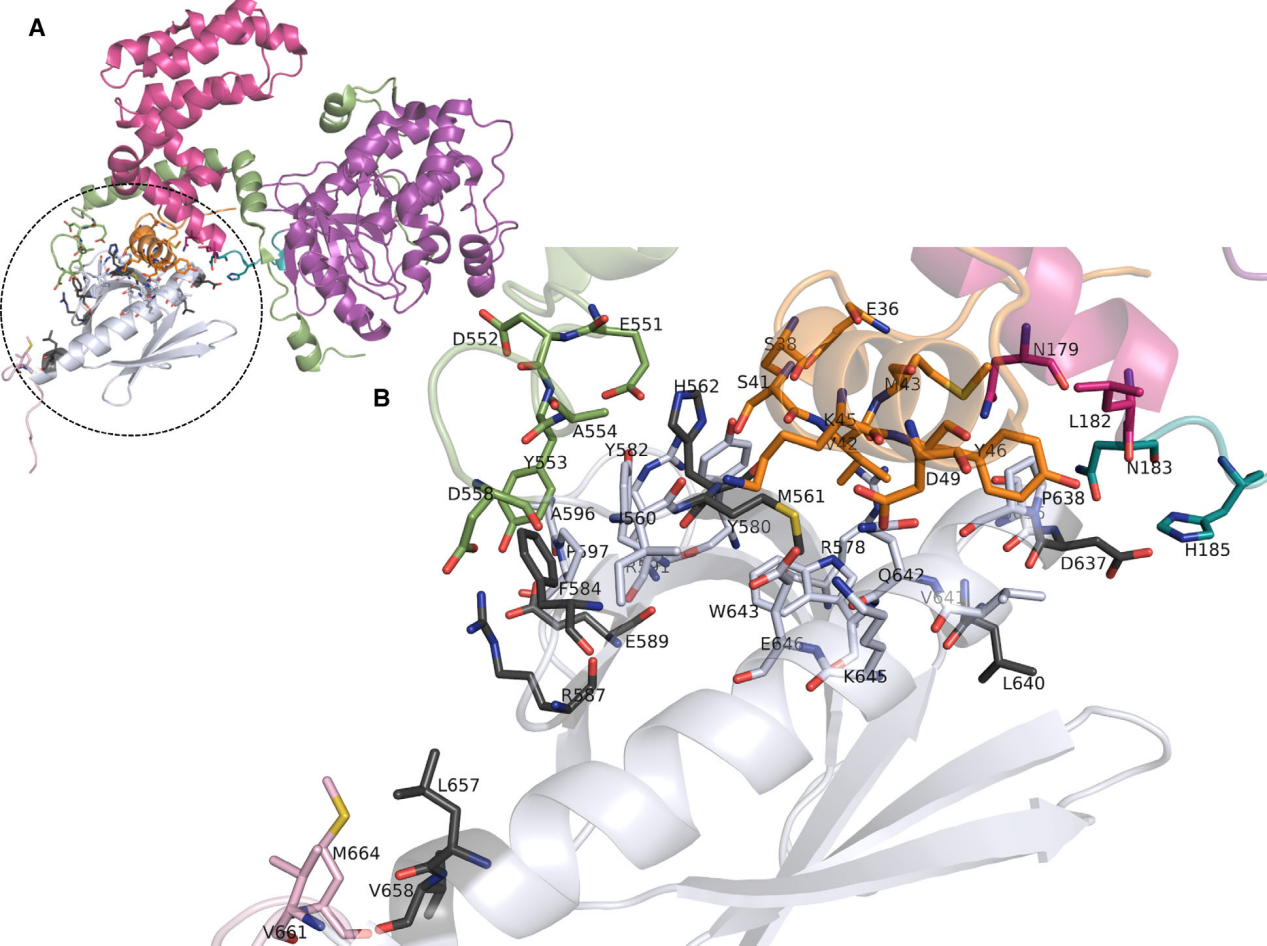
Interdomain interactions of GRK2 PH domain. (A) Interaction of PH domain with other regions of the GRK2 protein. PH domain is colored in white, N‐terminal helix (highlighted in orange), RGS‐kinase linker (highlighted in dark green), PH‐kinase linker (highlighted in green), RGS domain (highlighted in pink), and C‐terminal region (highlighted in light pink). (B) A zoomed in view of the interface. Interface residues shown in sticks, DA‐specific residues are highlighted in gray color.

Pleckstrin homology domain interacts with other regions of the GRK2 protein as shown in Fig. 4. In this case, PH domain interacts with different regions of GRK2 such as N‐terminal helix, RGS‐kinase linker, PH‐kinase linker, RGS domain, and C‐terminal regions and maintains its functional form.

**Figure 5:**
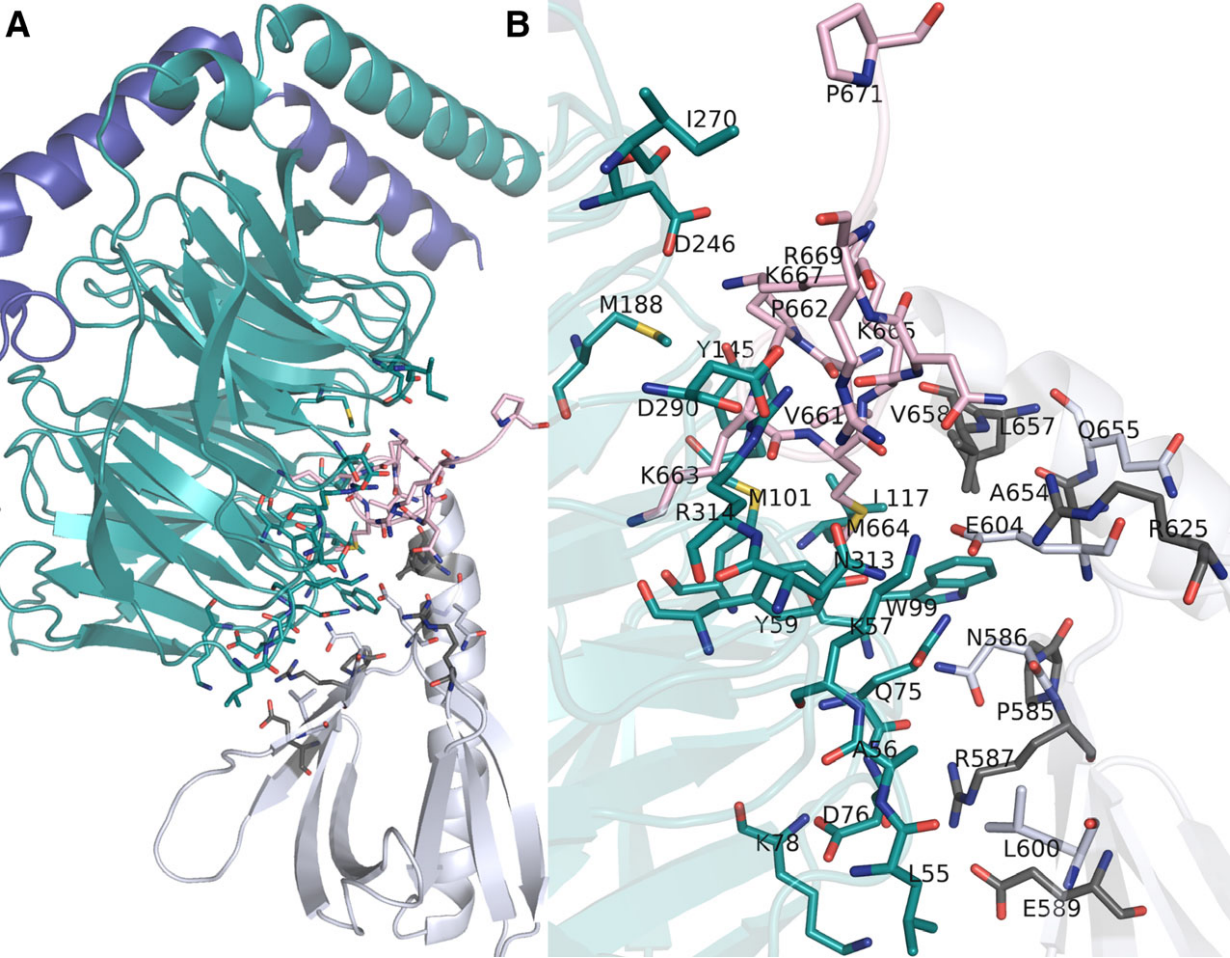
GRK2 PH domain interaction with Gβγ subunits. (A) PH domain interaction with Gβγ subunits, (B) a zoomed in view of the interface. PH domain is colored in white, and the Gβ protein is colored in green and blue. The interface residues were shown in sticks. DA‐specific residues are highlighted in gray.

Pleckstrin homology domain of GRK2 and its C‐terminal extension mediates interactions with Gβγ subunits (Fig. 5). Apart from involving in direct protein–protein interaction, there are certain residues that contribute to the conformation of interface through intradomain interactions with interface residues.

### DA‐specific residues that mediate phospho‐inositol binding in GRK2

As mentioned earlier, PtdIns (3,4) P2 and PtdIns (4,5) P2 are known to bind GRK2 PH domain. It is known that GRK2 PH domain has two binding pockets for phosphoinositide, where the loop1–loop3 forms one and the loop1–loop5 forms the other 21. Interestingly, 8 of the DA‐specific residues for phosphoinositide binding are in these loop regions. Residue Asp‐637, which is not a part of these loop regions, is known to play an important role in conferring phosphoinositide binding 21 (Fig. S5; Table 2).

## Conclusions

Pleckstrin homology domains are known to have a highly conserved fold and a general property of phospholipid binding and localization to the membrane by associating with βγ subunits of heterotrimeric G proteins. In the current work, two of the subfamilies of homologous PH domains were defined on the basis of their occurrence in two different DAs in two multidomain proteins, ARNO and GRK2. Though there are several residues that are conserved completely across these two subfamilies of PH domain, DA‐specific residues were identified in the two sets of PH domain sequences. We identified potential reasons for the DA‐specific conservation. We note that many of these residues in ARNO and GRK2 PH domains are involved in intraprotein interactions with domains and domain–domain linkers outside PH domain. This observation is consistent with the fact that ARNO and GRK2 have very different DAs. Further, some of the DA‐specific residues are found to form variable intraprotein interactions facilitating structural excursions among inactive, active, and partially active forms of ARNO. Some of the DA residues are also identified to confer specificity to specific phospholipids.

Our work provides a convenient general framework to understand the residue determinants of subfamily specific functional and structural features for subfamilies in other protein domain families too.

## Conflict of interest

The authors declare no conflict of interest.

## Author contributions

NN performed all the analyses of sequences and structures and wrote computer programs. RS and NS conceived the idea and designed the project. NN wrote the first draft of the manuscript. All the three authors agreed on the final version of the manuscript.

## Supporting information


**Fig. S1.** Multiple SA of PH domain regions of ARNO and GRK2 sequences.

**Fig. S2.** Two different conformers of ARNO in auto‐inhibited state.

**Fig. S3.** Phosphoinositide binding with ARNO PH domain.

**Fig. S4.** ARNO interaction with Arf6.

**Fig. S5.** Residues that confer phosphoinositide interaction in PH domain of GRK2.

**Appendix S1.** Perl scripts to search the Pfam sequence database for multi‐domain proteins of specific domain architecture.

## References

[1] Kalaivani R , Reema R and Srinivasan N (2018) Recognition of sites of functional specialisation in all known eukaryotic protein kinase families. PLoS Comput Biol 14, e1005975. 29438395 10.1371/journal.pcbi.1005975 PMC5826538

[2] Bhaskara RM , Padhi A and Srinivasan N (2014) Accurate prediction of interfacial residues in two‐domain proteins using evolutionary information: implications for three‐dimensional modeling. Proteins 82, 1219–1234. 24375512 10.1002/prot.24486

[3] Vishwanath S , de Brevern AG and Srinivasan N (2018) Same but not alike: Structure, flexibility and energetics of domains in multi‐domain proteins are influenced by the presence of other domains. PLoS Comput Biol 14, e1006008. 29432415 10.1371/journal.pcbi.1006008 PMC5825166

[4] Tyers M , Rachubinski RA , Stewart MI , Varrichio AM , Shorr RG , Haslam RJ and Harley CB (1988) Molecular cloning and expression of the major protein kinase C substrate of platelets. Nature 333, 470–473. 2897630 10.1038/333470a0

[5] Riddihough G (1994) More meanders and sandwiches. Nat Struct Biol 1, 755–757. 7634082 10.1038/nsb1194-755

[6] Scheffzek K and Welti S (2012) Pleckstrin homology (PH) like domains ‐ versatile modules in protein‐protein interaction platforms. FEBS Lett 586, 2662–2673. 22728242 10.1016/j.febslet.2012.06.006

[7] Berman HM , Westbrook J , Feng Z , Gilliland G , Bhat TN , Weissig H , Shindyalov IN and Bourne PE (2000) The protein data bank. Nucleic Acids Res 28, 235–242. 10592235 10.1093/nar/28.1.235 PMC102472

[8] Cherfils J and Zeghouf M (2013) Regulation of small GTPases by GEFs, GAPs, and GDIs. Physiol Rev 93, 269–309. 23303910 10.1152/physrev.00003.2012

[9] DiNitto JP and Lambright DG (2006) Membrane and juxtamembrane targeting by PH and PTB domains. Biochem Biophys Acta 1761, 850–867. 16807090 10.1016/j.bbalip.2006.04.008

[10] Lemmon MA (2004) Pleckstrin homology domains: not just for phosphoinositides. Biochem Soc Trans 32, 707–711. 15493994 10.1042/BST0320707

[11] DiNitto JP , Delprato A , Gabe Lee MT , Cronin TC , Huang S , Guilherme A , Czech MP and Lambright DG (2007) Structural basis and mechanism of autoregulation in 3‐phosphoinositide‐dependent Grp1 family Arf GTPase exchange factors. Mol Cell 28, 569–583. 18042453 10.1016/j.molcel.2007.09.017 PMC2156038

[12] Malaby AW , Das S , Chakravarthy S , Irving TC , Bilsel O and Lambright DG (2018) Structural dynamics control allosteric activation of cytohesin family Arf GTPase exchange factors. Structure 26, 106–117.e6. 29276036 10.1016/j.str.2017.11.019 PMC5752578

[13] Lietzke SE , Bose S , Cronin T , Klarlund J , Chawla A , Czech MP and Lambright DG (2000) Structural basis of 3‐phosphoinositide recognition by pleckstrin homology domains. Mol Cell 6, 385–394. 10983985 10.1016/s1097-2765(00)00038-1

[14] Klarlund JK , Tsiaras W , Holik JJ , Chawla A and Czech MP (2000) Distinct polyphosphoinositide binding selectivities for pleckstrin homology domains of GRP1‐like proteins based on diglycine versus triglycine motifs. J Biol Chem 275, 32816–32821. 10913124 10.1074/jbc.M002435200

[15] Macia E , Paris S and Chabre M (2000) Binding of the PH and polybasic C‐terminal domains of ARNO to phosphoinositides and to acidic lipids. Biochemistry 39, 5893–5901. 10801341 10.1021/bi992795w

[16] Nagel W , Schilcher P , Zeitlmann L and Kolanus W (1998) The PH domain and the polybasic c domain of cytohesin‐1 cooperate specifically in plasma membrane association and cellular function. Mol Biol Cell 9, 1981–1994. 9693361 10.1091/mbc.9.8.1981 PMC25450

[17] Santy LC , Frank SR , Hatfield JC and Casanova JE (1999) Regulation of ARNO nucleotide exchange by a PH domain electrostatic switch. Curr Biol 9, 1173–1176. 10531036 10.1016/S0960-9822(00)80019-6

[18] Carman CV and Benovic JL (1998) G‐protein‐coupled receptors: turn‐ons and turn‐offs. Curr Opin Neurobiol 8, 335–344. 9687355 10.1016/s0959-4388(98)80058-5

[19] Sterne‐Marr R and Benovic JL (1995) Regulation of G protein‐coupled receptors by receptor kinases and arrestins. Vitam Horm 51, 193–234. 7483322 10.1016/s0083-6729(08)61039-0

[20] Pitcher JA , Inglese J , Higgins JB , Arriza JL , Casey PJ , Kim C , Benovic JL , Kwatra MM , Caron MG and Lefkowitz RJ (1992) Role of beta gamma subunits of G proteins in targeting the beta‐adrenergic receptor kinase to membrane‐bound receptors. Science 257, 1264–1267. 1325672 10.1126/science.1325672

[21] Carman CV , Barak LS , Chen C , Liu‐Chen LY , Onorato JJ , Kennedy SP , Caron MG and Benovic JL (2000) Mutational analysis of Gbetagamma and phospholipid interaction with G protein‐coupled receptor kinase 2. J Biol Chem 275, 10443–10452. 10744734 10.1074/jbc.275.14.10443

[22] Waldschmidt HV , Homan KT , Cato MC , Cruz‐Rodriguez O , Cannavo A , Wilson MW , Song J , Cheung JY , Koch WJ , Tesmer JJ *et al* (2017) Structure‐based design of highly selective and potent G protein‐coupled receptor kinase 2 inhibitors based on paroxetine. J Med Chem 60, 3052–3069. 28323425 10.1021/acs.jmedchem.7b00112 PMC5641445

[23] Muchmore SW , Sattler M , Liang H , Meadows RP , Harlan JE , Yoon HS , Nettesheim D , Chang BS , Thompson CB , Wong SL * et al* (1996) X‐ray and NMR structure of human Bcl‐xL, an inhibitor of programmed cell death. Nature 381, 335–341. 8692274 10.1038/381335a0

[24] Onorato JJ , Gillis ME , Liu Y , Benovic JL and Ruoho AE (1995) The beta‐adrenergic receptor kinase (GRK2) is regulated by phospholipids. J Biol Chem 270, 21346–21353. 7673171 10.1074/jbc.270.36.21346

[25] DebBurman SK , Ptasienski J , Boetticher E , Lomasney JW , Benovic JL and Hosey MM (1995) Lipid‐mediated regulation of G protein‐coupled receptor kinases 2 and 3. J Biol Chem 270, 5742–5747. 7890702 10.1074/jbc.270.11.5742

[26] DebBurman SK , Ptasienski J , Benovic JL and Hosey MM (1996) G protein‐coupled receptor kinase GRK2 is a phospholipid‐dependent enzyme that can be conditionally activated by G protein betagamma subunits. J Biol Chem 271, 22552–22562. 8798423 10.1074/jbc.271.37.22552

[27] Pitcher JA , Touhara K , Payne ES and Lefkowitz RJ (1995) Pleckstrin homology domain‐mediated membrane association and activation of the beta‐adrenergic receptor kinase requires coordinate interaction with G beta gamma subunits and lipid. J Biol Chem 270, 11707–11710. 7744811 10.1074/jbc.270.20.11707

[28] Fushman D , Najmabadi‐Haske T , Cahill S , Zheng J , LeVine H 3rd and Cowburn D (1998) The solution structure and dynamics of the pleckstrin homology domain of G protein‐coupled receptor kinase 2 (beta‐adrenergic receptor kinase 1). A binding partner of Gbetagamma subunits. J Biol Chem 273, 2835–2843. 9446593 10.1074/jbc.273.5.2835

[29] Sonnhammer EL , Eddy SR and Durbin R (1997) Pfam: a comprehensive database of protein domain families based on seed alignments. Proteins 28, 405–420. 9223186 10.1002/(sici)1097-0134(199707)28:3<405::aid-prot10>3.0.co;2-l

[30] UniProt C (2019) UniProt: a worldwide hub of protein knowledge. Nucleic Acids Res 47, D506–D515. 30395287 10.1093/nar/gky1049 PMC6323992

[31] Fu L , Niu B , Zhu Z , Wu S and Li W (2012) CD‐HIT: accelerated for clustering the next‐generation sequencing data. Bioinformatics 28, 3150–3152. 23060610 10.1093/bioinformatics/bts565 PMC3516142

[32] Katoh K , Rozewicki J and Yamada KD (2017) MAFFT online service: multiple sequence alignment, interactive sequence choice and visualization. Brief Bioinform. 10.1093/bib/bbx108..> 10.1093/bib/bbx108.. PMC6781576 28968734

[33] Waterhouse AM , Procter JB , Martin DM , Clamp M and Barton GJ (2009) Jalview Version 2–a multiple sequence alignment editor and analysis workbench. Bioinformatics 25, 1189–1191. 19151095 10.1093/bioinformatics/btp033 PMC2672624

[34] Tina KG , Bhadra R and Srinivasan N (2007) PIC: protein interactions calculator. Nucleic Acids Res 35, W473–476. 17584791 10.1093/nar/gkm423 PMC1933215

[35] Hubbard SJ and Thornton JM (1993) Naccess. Computer Program, Department of Biochemistry and Molecular Biology, University College London.

[36] The PyMOL Molecular Graphics System, Version 2.0 Schröodinger, LLC..

