# Supplementary material for: Specialized structural and functional roles of residues selectively conserved in subfamilies of the pleckstrin homology domain family
Source: FEBS Open Bio. 2019 Sep 30;9(11):1848–59. doi: 10.1002/2211-5463.12725 (PMC6823287; doi:10.1002/2211-5463.12725)
Supplement: Supplementary file 1 — Fig. S1. Multiple SA of PH domain regions of ARNO and GRK2 sequences. [file FEB4-9-1848-s001.pdf]

Figure-S1:

Multiple sequence alignment of PH domain regions of ARNO and GRK2 sequences. 88 ARNO sequences 21 GRK2 sequences

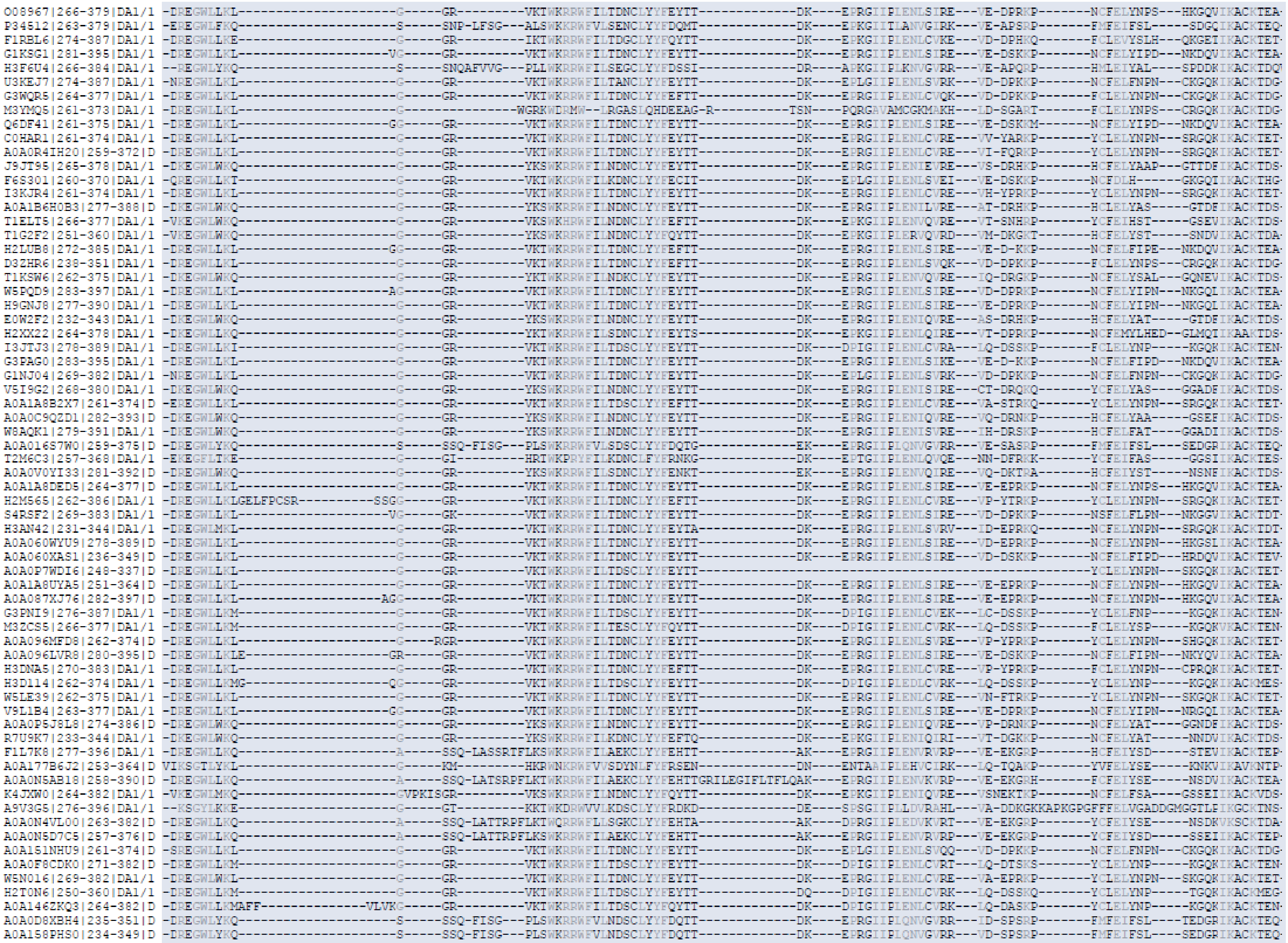

```
A0A0N5DZN7|259-370|D -DRQGWLWKQ---------------------G-----GR---------YRSWKRRWFVLSDNCLYYFENNG------------DK----EPRGIIPLENVQVRQ---VV-DKTRA-------YSFEIYST-----NAGLIKACKTDS·
A0A131Z502|285-396|D -DREGWLWKQ---------------------G-----GR---------YKSWKRRWFILNDNCLYYFEYTT------------DK----EPRGIIPLENVQVKE---VQ-DRHKP-------NCFELYSV-----GSELIKACKTDS·
A0A077YZD5|260-371|D -DREGWLWKQ---------------------G-----GR---------YRSWKRRWFVLSDNCLYYFENTG------------DK----EPRGIIPLENVQVRQ---VV-DRTRA-------YGFEIYST-----NAGLIKACKTDS·
K1QXG2|263-376|DA1/1 -DKEGWLCKQ---------------------G-----GR---------IKNWKRRWFILNDNCLYYFQFTT------------DK----EPKGIIPLENISIREV-PVD-KSNKP-------FSFELFAT-----GQDIIKACKVDK·
L5MI93|206-319|DA1/1 -DREGWLLKL---------------------G-----GR---------VKTWKRRWFILTDNCLYYFEYTT------------DK----EPRGIIPLENLSIRE---VE-DSKKP-------NCFELYIPD---SKDQVIKACKTEA·
A0A0J9R6N8|281-393|D -DKEGWLWKQ---------------------G-----GR---------YKSWKRRWFILNDNCLYYFEYTT------------DK----EPRGIIPLENISVRE---IH-DRSKP-------HCFELFAT----GGADIIKACKTDS·
H2UST7|262-394|DA1/1 -DREGWLLKLDT--PSHRMSPLSTLVRSAVSG-----GR---------VKTWKRRWFILTDNCLYYFEYTT------------DK----EPRGIIPLENLSIRE---VE-DPRKP-------NCFELYIPN---NRGQLIKACKTEA·
H2VB39|265-379|DA1/1 -DREGWLLKL--------------------GS-----GR---------VKTWKRRWFILTDNCLYYFEYTT------------DK----EPRGIIPLENLSIRE---VE-DSKKP-------NCFELFIPD---HKDQVIKACKTEA·
F1RZ72|262-376|DA1/1 -DREGWLLKL--------------------AG-----GR---------VKTWKRRWFILTDNCLYYFEYTT------------DK----EPRGIIPLENLGIRE---VE-DSKKP-------NCFELYIPD---SKDQVIKACKTEA·
G1SWB5|267-380|DA1/1 -DREGWLLKL---------------------GTAPGRGE---------LGAWVLGW----ENVLVCFSYSL------------DK----EPRGIIPLENLSIRE---VD-DPRKP-------NCFELYIPN---NKGQLIKACKKEA·
L5KFZ4|283-396|DA1/1 -DREGWLLKL---------------------G-----GR---------VKTWKRRWFILTDSCLYYFEFTT------------DK----EPRGIIPLENLSVQK---VD-DPKKP-------FCLELYNPS---CRGQKIKACKTDG·
A0A0K0EDG6|263-373|D -DKKGFLWKK---------------------G-----S----------YKTWKKRWFILSGKCLYYFENQG------------ET----DPRGIIPLENVKVRF---VE-EKNKQ-------HVFEIFSS-----TSEVIKACKTET·
Q7PPK6|250-362|DA1/1 -DKEGWLWKQ---------------------G-----GR---------YKSWKRRWFILNDNCLYYFEYTT------------DK----EPRGIIPLENIAVRE---VT-DRSKP-------HCFELHAS----GGADIIKACKTDS·
A0A0D2WVY4|271-383|D -EREGWLTKQ---------------------G-----GR---------KKNWRKRWFVLTGNCLYYFKETA------------DP----HPLGIIPLEDLRVRD---VSVASKRA-------HCFEIYNA-----TDGFIKACKTSS·
C3Y8Z8|261-375|DA1/1 -DKEGWLLKQ---------------------G-----GR---------HKTWKRRWFILTDNCLYYFEYTT------------DK----EPRGIIPLENLQVRL---VE-DSKKP-------NCFELYPSEA--NSKAVIKACKTDS·
A0A0K2U7R3|265-376|D -DREGWLWKQ---------------------G-----GR---------YKSWKRRWFILNDNCLYYFEFTT------------DK----EPRGIIPLENIRVRE---VG-DRNKS-------HCFELFSA-----GNEVIKACKTDS·
T1IR78|273-384|DA1/1 -DREGWLWKQ---------------------G-----GR---------YKSWKRRWFILNDNCLYYFEYTT------------DK----EPRGIIPLENIQIRE---VN-DRSKP-------NCFELFAS-----GSDVIKACKTDS·
D1LWZ6|270-384|DA1/1 -DKEGWLLKQ---------------------G-----GR---------YKNWKRRWFILTDNCLYYFEYTT------------DK----EPRGIIPLENLNVKDN-DIK-ESKKP-------FCFELFSS----DGNSVIKACKTDA·
A0A0N4U532|260-379|D -DHEGWLLKQ---------------------A-----SSQ-LATSRPFLKSWKRRWFILAEKCLYYFEHTT------------AK----EPRGIIPLENVRVRQ---IE-EKGRP-------YCFEIYSD-----NTEVIKACKTEA·
A0A0P4W3P2|268-379|D -DREGWLMKQ---------------------G-----GR---------YKSWKRRWFILNDNCLYYFEYTT------------DK----EPRGIIPLENIEVRE---VQ-DRNKP-------NCFELYAT-----STDFIKACKTDS·
T1PAQ9|281-393|DA1/1 -DKEGWLWKQ---------------------G-----GR---------YKSWKRRWFILNDNCLYYFEYTT------------DK----EPRGIIPLENICVRE---IH-DRSKP-------NCFELFAT----GGAEIIKACKTDS·
P25098|560-650|DA2/1 -IMHGYMSKM---------------------G-----NP--------FLTQWQRRYFYLFPNRLEWRGEGE------------------APQSLLTMEEIQSVEE--TQI-KERK--------CLLIKIR----------------G·
P26819|560-647|DA2/1 -IMHGYMLKL---------------------G-----NP--------FLTQWQRRYFYLFPNRLEWRGEGE------------------SRQNLLTMEQIMSVEE--TQI-KDRK--------CILIRVK----------------G·
P32865|560-656|DA2/1 --LHGYIKKL---------------------G-----GS--------FASLWQTKYAKLYPNRLELHSESG------------NN-----KPELIFMDQVEDISSDFILH-KNEN--------CIQIRIN-------------DGTR·
F1RUX3|523-613|DA2/1 -IMHGYMSKM---------------------G-----NP--------FLTQWQRRYFYLFPNRLEWRGEGE------------------APQSLLTMEEIQSVEE--TQI-KERK--------CLLIKIR----------------G·
G1R853|530-619|DA2/1 -IMHGYMLKL---------------------G-----NP--------FLTQWQRRYFYLFPNRLEWRGEGE------------------SRQNLLTMEQILSVEE--TQI-KDKK--------CILFRIK----------------G·
M3YQE1|569-657|DA2/1 -IMHGYMLKL---------------------G-----NP--------FLTQWQRRYFYLFPNRLEWRGEGE------------------SRQNLLTMEQILSVEE--TQI-KDKK--------CILIRIK----------------G·
F7DR73|561-649|DA2/1 --LHGYMLKL---------------------G-----NP--------FLTQWQRRYFYLFPNRLEWRGEGE------------------SRQNLVTMEQVVSVEE--TQI-KDKK--------CILIRVK----------------G·
A0A1B0B9X5|560-656|D --LHGYIKKY---------------------G-----GS--------FASLWQTKYAKLYPNRLELHSESG------------NN-----KPELIFMDQIEDISSDFVPY-KGEQ--------CIQIRVN-------------DGSR·
C3YDG6|559-650|DA2/1 -IIHGHLLKL---------------------G-----GP--------FLTAWQKRYFWLFPNRLEWQGEAE------------------KGNNVLVIESVLAVEE--TTV-KGNK--------CIQLKVK----------------G·
T1JT63|559-655|DA2/1 --LHGYLKKL---------------------G-----GP--------FASNWQTRYGKLYPNRLELHSESV------------NT-----KPELIFMDQIEDISPEYVQI-KSEN--------SIILKLK------------GEPGK·
X1X0Z1|561-655|DA2/1 --LHGYIKKL---------------------G-----GP--------FASAWQTRYAKLYPNRLELHSDSG------------SV-----KTEVITLDQVLEVSPELVIV-KNEQ--------CIVIRMK-------------DG-K·
A0A158R463|563-660|D VIIHGYIKKL---------------------G-----GP--------FASAWQTKYGKLYPSRLELYSESL------------SG-----RPELVFMDQIEDVGSELVSF-KGET--------TIVIKLK-------------EGFK·
U3FBJ7|560-650|DA2/1 -IMHGYMSKL---------------------G-----NP--------FLTQWQRRYFYLFPNRLEWRGEGE------------------SPQSLLTMEEIQSVEE--TQV-KDRK--------CILIKIR----------------G·
Q9U756|561-654|DA2/1 -IIHGYIKKL---------------------G-----GP--------FTSAWQTRYAKLYPNRVELHSESS------------SG-----KPELIFMDQLDDVSSDFVQI-KGEN--------CIVIKT-----------------K·
A0A0V1EVP4|557-653|D VIIHGYIKKL---------------------G-----GP--------FTTAWQQRYGKLYPSRLELYPESL------------SG-----KPELYFMDQIEDVSADLRPV-KGEQ--------SIVIRFR-------------DGTK·
A0A151NSD8|543-633|D -IMHGYMLKL---------------------G-----NP--------FLTQWQRRYFYLFPNRLEWRGEGE------------------SRQNLLTMEQIVSVEE--TQI-KDKK--------CILIRIK----------------G·
W5M5B5|560-650|DA2/1 -IMHGYMLKL---------------------G-----NP--------FLTQWQRRYFYLFPNRVEWRGEGE------------------SRQNLLTMEQIVSVEE--TQV-KEKK--------CILIRIK----------------G·
H2YCX8|557-647|DA2/1 --LHGIMYKL---------------------G-----GPL-------ALTTWQKRYCYLYPNRLEWKDYDG-------------------SKNLVVFESVTMIKE--GQA-KGMK-------YCLFISTR---------------ES·
O97020|557-653|DA2/1 -IVEGEILKL---------------------G-----GP--------FLNSWQKRHLRLYPNRLEFYQKNR------------DGGIQKNKVELLTMYDIKEVCQEFQKLNKTDN--------CIVMVLK----------------N·
L5LZP7|548-636|DA2/1 -IMHGYMLKL---------------------G-----NP--------FLTQWQRRYFYLFPNRLEWRGEGE------------------SRQNLLTMEQIMSVEE--TQF-KDKK--------CILIRIK----------------G·
A0A0N5E479|558-654|D VIIHGYIKKL---------------------G-----GP--------FTSAWQQKYGKLYPSRLELYSESL------------SA-----KPELLFMDQIEDVAAELRSV-KGEQ--------SIIVKYR-------------DTGK·
```
