# Supplementary figures and images for: Specialized structural and functional roles of residues selectively conserved in subfamilies of the pleckstrin homology domain family
Source: FEBS Open Bio. 2019 Sep 30;9(11):1848–59. doi: 10.1002/2211-5463.12725 (PMC6823287; doi:10.1002/2211-5463.12725)

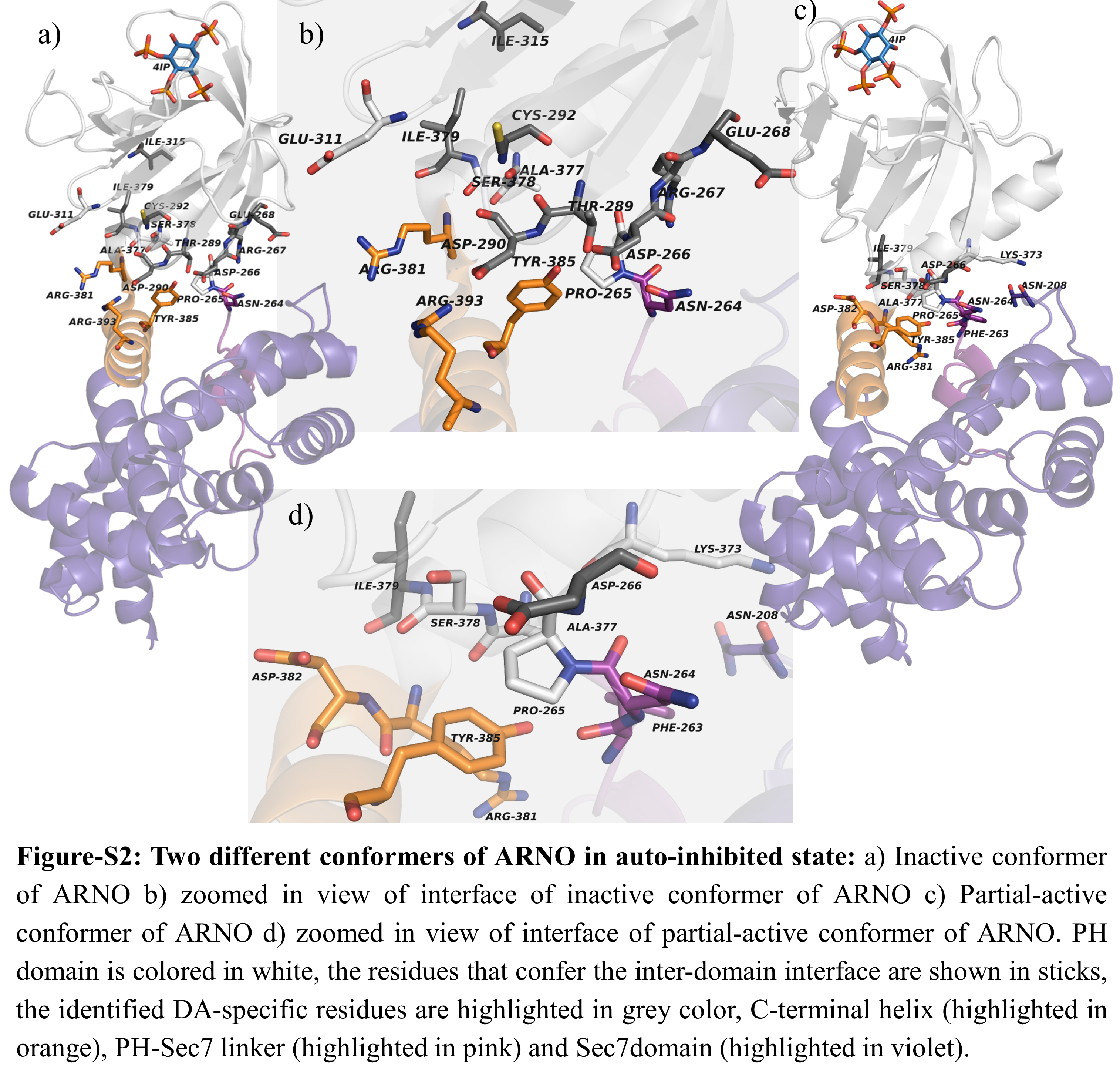

Supplement: Supplementary file 2 — Fig. S2. Two different conformers of ARNO in auto‐inhibited state. [file FEB4-9-1848-s002.tif]

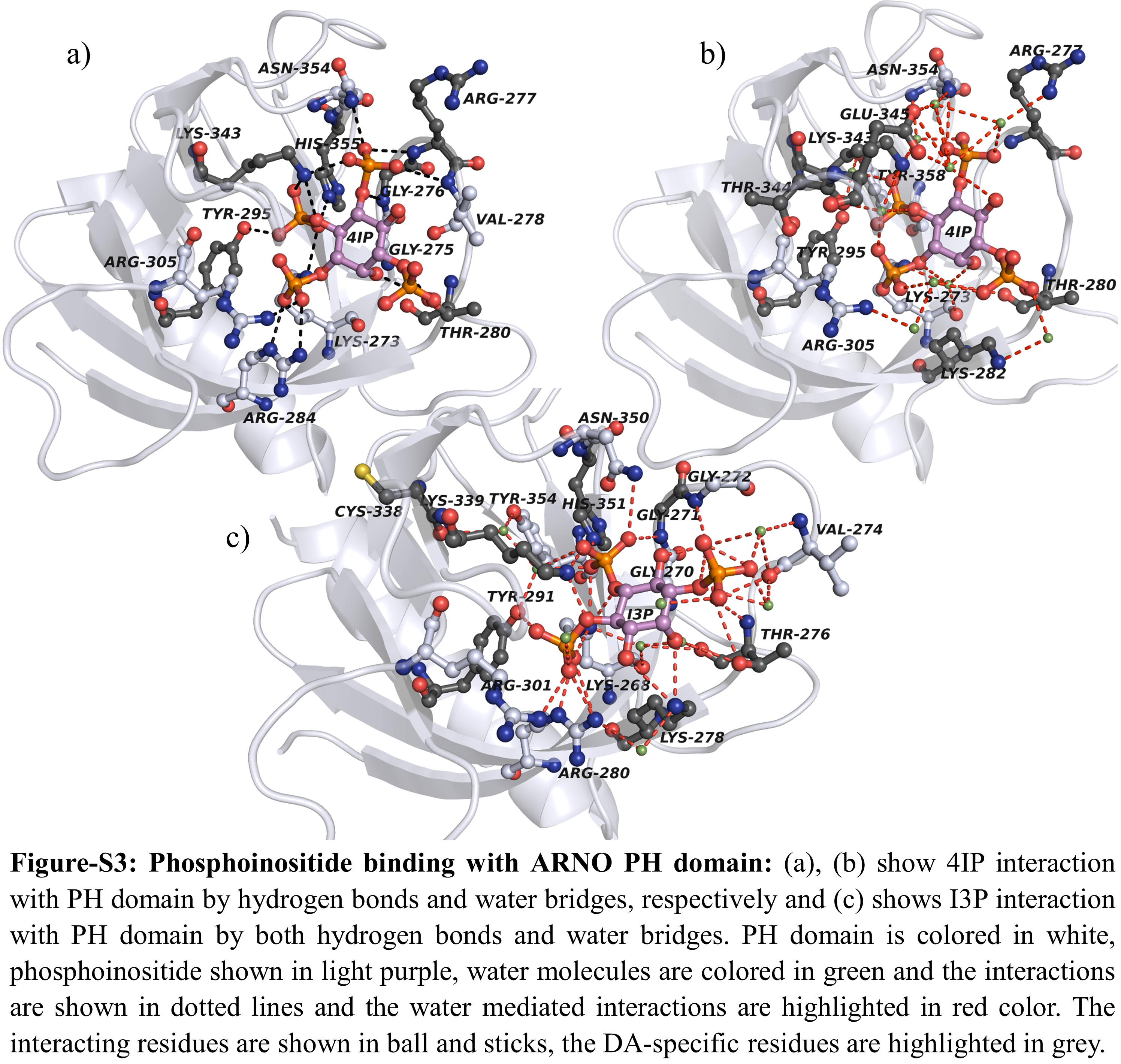

Supplement: Supplementary file 3 — Fig. S3. Phosphoinositide binding with ARNO PH domain. [file FEB4-9-1848-s003.tif]

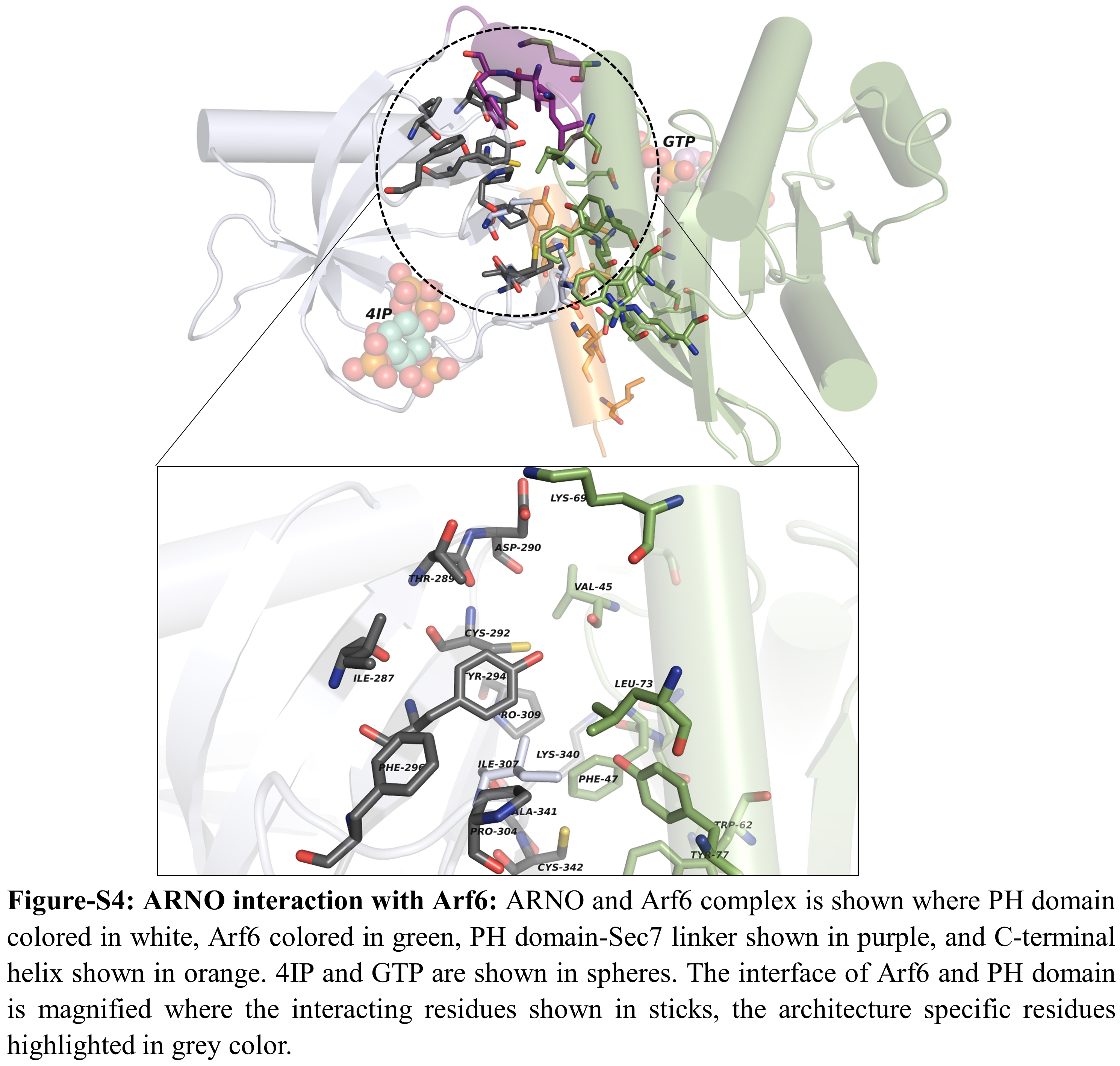

Supplement: Supplementary file 4 — Fig. S4. ARNO interaction with Arf6. [file FEB4-9-1848-s004.tif]

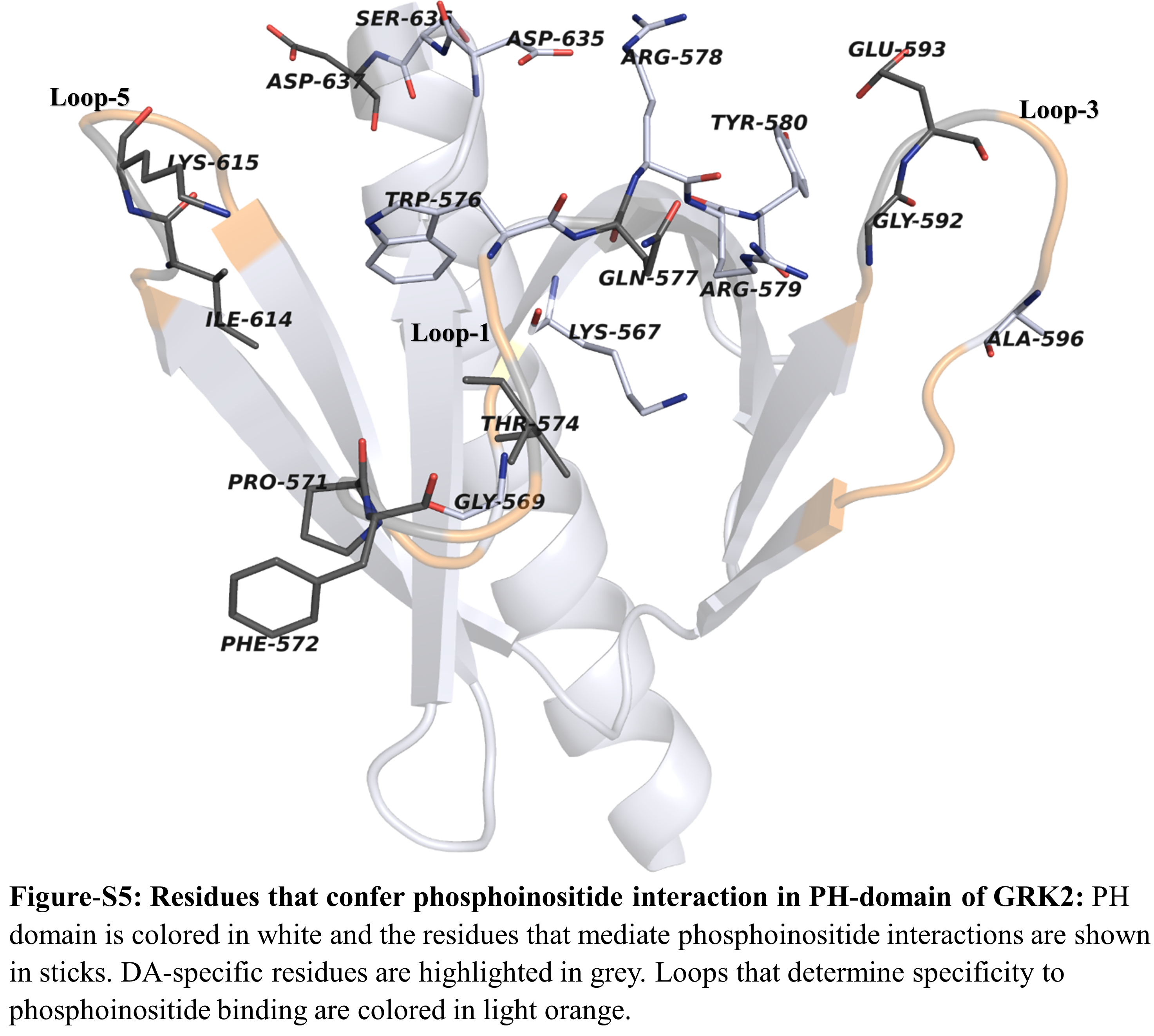

Supplement: Supplementary file 5 — Fig. S5. Residues that confer phosphoinositide interaction in PH domain of GRK2. [file FEB4-9-1848-s005.tif]
